# Acute atrial fibrillation and flutter treated electively: rationale and design of the randomized controlled AFFELECT trial

**DOI:** 10.1093/ehjopen/oeag060

**Published:** 2026-04-13

**Authors:** Jani Rankinen, Leo-Pekka Lyytikäinen, Juuso Järventie, Markus Hautamäki, Anna Numminen, Matilda Hurskainen, Markku Rantanen, Antti Muuronen, Juho Tynkkynen, Jussi Hernesniemi

**Affiliations:** Tampere Heart Hospital, Tampere University Hospital, Elämänaukio 1, 33520 Tampere, Finland; Faculty of Medicine and Health Technology and Finnish Cardiovascular Research Center, Tampere University, Arvo Ylpön katu 34, 33520 Tampere, Finland; Tampere Heart Hospital, Tampere University Hospital, Elämänaukio 1, 33520 Tampere, Finland; Faculty of Medicine and Health Technology and Finnish Cardiovascular Research Center, Tampere University, Arvo Ylpön katu 34, 33520 Tampere, Finland; Tampere University Hospital, Elämänaukio 2, 33520 Tampere, Finland; Faculty of Medicine and Health Technology and Finnish Cardiovascular Research Center, Tampere University, Arvo Ylpön katu 34, 33520 Tampere, Finland; Tampere Heart Hospital, Tampere University Hospital, Elämänaukio 1, 33520 Tampere, Finland; Faculty of Medicine and Health Technology and Finnish Cardiovascular Research Center, Tampere University, Arvo Ylpön katu 34, 33520 Tampere, Finland; Faculty of Medicine and Health Technology and Finnish Cardiovascular Research Center, Tampere University, Arvo Ylpön katu 34, 33520 Tampere, Finland; Tampere Heart Hospital, Tampere University Hospital, Elämänaukio 1, 33520 Tampere, Finland; Tampere Heart Hospital, Tampere University Hospital, Elämänaukio 1, 33520 Tampere, Finland; Centre for Vascular Surgery and Interventional Radiology, Tampere University Hospital, Elämänaukio 2, 33520 Tampere, Finland; Tampere Heart Hospital, Tampere University Hospital, Elämänaukio 1, 33520 Tampere, Finland; Faculty of Medicine and Health Technology and Finnish Cardiovascular Research Center, Tampere University, Arvo Ylpön katu 34, 33520 Tampere, Finland

**Keywords:** Atrial fibrillation, Atrial flutter, Rhythm control, Cardioversion

## Abstract

**Aims:**

Cardioversion (CV) is commonly used in the emergency department (ED) to treat recent-onset atrial fibrillation (AF) or flutter (AFL).

**Design:**

The AFFELECT trial (NCT04267159) is an investigator-initiated, prospective, unblinded randomized controlled non-inferiority trial comparing experimental delayed rhythm control (elective CV performed within 5–9 days after the index visit) to standard acute rhythm control (CV performed in ED) in patients with recent-onset (duration <48 h) symptomatic AF/AFL suitable for rhythm control. A total of 500 patients are randomized in a 2:3 ratio to the acute and delayed groups, respectively, accounting for a possible one-third unplanned early CV rate in the delayed group due to higher symptom burden. Unplanned early CV means that patients with unbearable symptoms are offered the option for an earlier CV (before the 5–9 days target timeline) if needed. Patients randomized to delayed group are discharged immediately after adequate heart rate control (heart rate <110 bpm) and anticoagulation and are scheduled an appointment for delayed CV at a cardiology outpatient clinic (in transoesophageal echocardiography guidance if required). Patients randomized to acute CV undergo cardioversion in the ED within 48 h of arrhythmia onset and are assigned to a cardiologic outpatient clinic visit also within 5–9 days. The primary end-point is the presence of sinus rhythm on electrocardiogram at 4 weeks after the outpatient clinic visit.

**Conclusion:**

The AFFELECT trial tests whether delayed management of recent-onset AF/AFL is a non-inferior alternative to acute CV, aiming to reduce ED burden, number of needed CVs, and redirect care to specialized arrhythmia units.

## Graphical Abstract

**Graphical Abstract:**
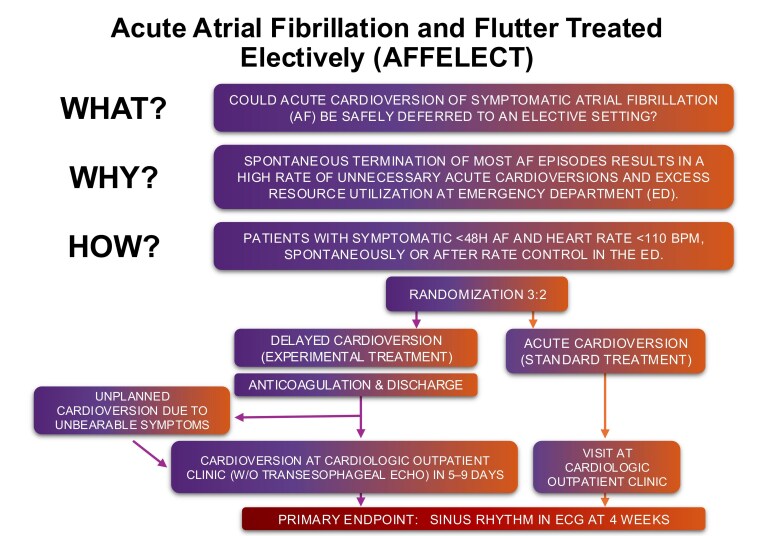
For image description, please refer to the figure legend and surrounding text.

## Introduction

Treatment of atrial fibrillation (AF) and atrial flutter (AFL) places substantial demands on healthcare resources and incurs significant costs.^1–5^ Urgent care of atrial arrhythmias encompasses conditions associated with chronic arrhythmias, such as heart failure or symptoms related to insufficient rate control, as well as cardioembolic events like ischaemic strokes. However, paroxysmal attacks of AF or AFL remain the most common reason for emergency department (ED) visits in this patient group.^6,7^

Symptomatic recent-onset (<48 h) attacks of AF or AFL are usually treated by cardioversion (CV) in the ED.^6,8^ In patients with no haemodynamical compromise, early rhythm control therapy is usually applied with the aim of improving quality of life.^9^ Sinus rhythm (SR) is restored either by electrical defibrillation of the heart or by pharmacological agents.^8,9^ The conventional rationale for CVs in the ED setting is acute symptom relief and also the fear of the possible deleterious causal effects of prolonged arrhythmia for long-term rhythm control.^9–11^ Furthermore, before the widespread use of direct anticoagulants, a considerable portion of patients were not permanently anticoagulated making rapid rhythm control a more desirable option due to its lower thromboembolic risk when compared to delayed rhythm control.^12^ This was further exacerbated by the unstable anticoagulative levels related to the use of vitamin K-antagonist, usually warfarin.^13^

The concern with active early CV of these atrial arrhythmias is that it may be unnecessary. Historical data^14,15^ and a recent randomized controlled trial^16^ shows that 60–70% of patients revert to normal SR spontaneously within days or weeks without any active treatment. Furthermore, there is no clear evidence that opting for early rhythm control reduces long-term AF/AFL burden or improves quality of life.^17,18^ Implementation of early rhythm control often results in frequent ED visits^16^ and electrical CV procedures require anaesthesia and medical CV requires relatively long follow-up time in the ED. For the high volume of these procedures, the overall costs are high.^5^

Previously in the RACE 7 ACWAS Trial, a delay within the first 48 h of arrhythmia onset was found non-inferior to acute CV in ED in respect to success in maintenance of sinus rhythm at 1 month after ED visit.^16^ However, there are no previous studies about the effects on rhythm control success for delaying rhythm control for longer periods of time. If the implementation of rhythm control in haemodynamically stable patients could be delayed to an outpatient setting and transferred to an elective setting, the number of ED visits and CV procedures could reduce dramatically and the treatment of patients with recurring arrhythmia could be directed to dedicated units specialized in the treatment of AF and AFL.

## The purpose of the study

The purpose of the AFFELECT trial is to test the hypothesis that delayed CV (elective approach) is non-inferior to acute CV (standard treatment) in rhythm control of recent-onset (<48 h) AF or AFL in respect to restoration of SR. Secondary aims are observing how many patients randomized to the elective approach require early CV due to a high symptom burden, the number of needed CVs and initiated antiarrhythmic treatments, duration of the ED stay, hospitalizations, and overall symptom burden of patients in the first month.

## Methods

### Overall design

The AFFELECT trial is a prospective, multicentre, investigator-initiated, unblinded randomized controlled two-arm clinical trial comparing two different approaches of rhythm control in recent-onset (duration <48 h) AF and AFL. Delayed rhythm control (elective CV performed within 5–9 days after ED visit) is compared to acute CV performed in ED in a non-inferiority setting. All patients over 18 years of age with recent-onset AF or AFL are considered for participation regardless of the AF/AFL classification (first ever or repeated event, paroxysmal or persistent) or status of prior anticoagulation treatment. Each patient can participate only once in the trial. All participants are required to give written, informed consent.

The study is performed in Tampere Heart Hospital (Tampere) and Central Hospital of Kanta-Häme (Hämeenlinna), and Central Finland Central Hospital (Jyväskylä). These centres are sole providers of acute care in rhythm control for acute atrial arrhythmias in their respective geographical regions in Finland. All centres are the exclusive providers of acute cardiology care within their regions. Patient recruitment started in February 2020 and is ongoing.

**Figure 1:**
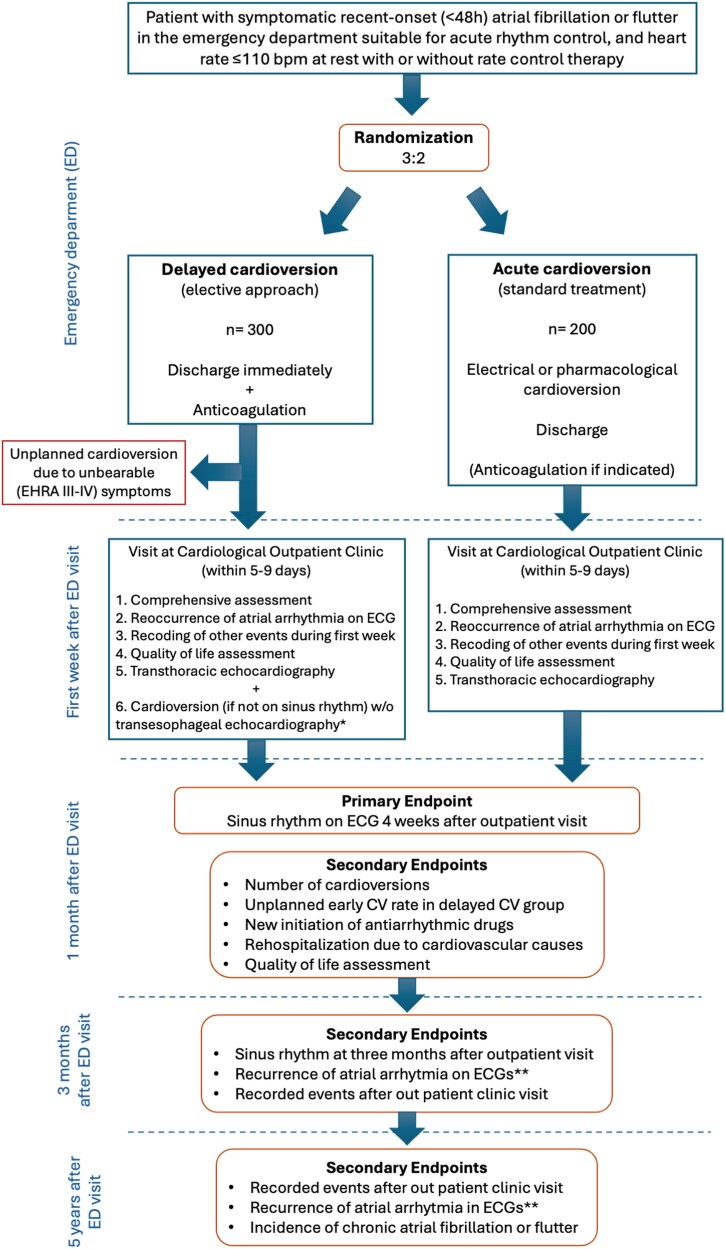
Flow chart of the AFFELECT trial. *if no previous uninterrupted anticoagulation for ≥3 weeks. **ECGs taken for clinical indication.

The trial protocol was approved by the ethics committee of Pirkanmaa Hospital district (ETL R19113) and by the institutional review boards of each participating centre. Local patient advocacy group (organization) for patients with heart diseases (Pirkanmaan Sydänliitto, Tampere, Finland) was consulted in the design phase of the trial. The trial is monitored by an independent monitor contracted from the Research Services of the hospital district of Pirkanmaa (The Wellbeing Services County of Pirkanmaa, Tampere, Finland). The AFFELECT trial (NCT04267159) is registered at ClinicalTrial.gov. The overall study protocol is depicted in a flow chart in *Figure 1*. The data can be made available upon reasonable request pending the authorization of the study steering committee.

### Patient eligibility

Patients with recent-onset AF/AFL (<48 h) who are suitable for acute rhythm control by CV as first-line treatment are screened for eligibility of participating in the study. These include both patients arriving to the ED directly and by referral. Patients considered for the study must be suitable for both acute and delayed rhythm control. The inclusion and exclusion criteria are described in *Table 1*. Possible high heart rate (>110 bpm) is acutely controlled before the final eligibility assessment, primarily using betablockers (usually bisoprolol), in accordance with guidelines established by the European Society of Cardiology (ESC)^9^ and American College of Cardiology/American Heart Association.^19^

**Table 1:** Inclusion and exclusion criteria

Inclusion criteria	Exclusion criteria
Age 18 years or older	Cardioversion not possible within 48 h if randomized to acute cardioversion
Recent-onset AF or AFL (<48 h)	Haemodynamically unstable: mean arterial pressure <60 mmHg
Planned acute rhythm control (acute CV) for the arrhythmia^a^ Heart rate ≤110 beats/min at rest either w/o medication after adequate rate control therapy	Cardioversion needed for some other somatic cause (for example poor clinical condition due to some other somatic cause requiring immediate restoration of sinus rhythm)^a^ Major co-existing illness predisposing to AF/AFL, including decompensated heart failure, ongoing myocardial infarction or hospitalization due to heart failure within 1 month of the ED visit, or severe valvular heart disease requiring urgent treatment.
	Anticoagulation not possible or safe due to poor co-operation or for clinical condition (for example chronic alcoholism, severe liver dysfunction, or history of severe spontaneous bleeding or bleeding under anticoagulation).
	Mitral stenosis or mechanical heart valve (any position)
	Need for prolonged hospitalization (over 24 h) due to any cause.
	Exceptionally high risk of thromboembolism (e.g. history of thromboembolism regardless of anticoagulation)
	Transoesophageal echocardiogram is contraindicated
	Participation unsuitable due to any somatic or psychiatric condition^a^
	Unable or unwilling to provide written informed consent

AF, atrial fibrillation; AFL, atrial flutter; ED, emergency department.

^a^At the discretion of the attending physician.

If the arrhythmia onset time is not clear due to mild or non-existent symptoms and the time from arrhythmia onset to CV cannot be determined unequivocally the patient is not eligible for participation regardless of anticoagulation status. Haemodynamically unstable patients, as well as those with other major complicating acute illnesses, are excluded from the study. If randomized to acute CV group, the procedure must be performed within 48 h of arrhythmia onset. Subjects must be suitable for discharge from the ED despite the treatment group. Furthermore, if rate control strategy is decided instead of acute rhythm control in the ED as a first-line treatment strategy, the patient is not eligible.

The treating physician in the ED always has the final authority to determine the patient’s eligibility for the study and remains responsible for the patient’s care. Eligible and willing patients are required to give informed consent which is received by study personnel trained in the protocol.

### Randomization

Patients are randomized either to acute CV (standard treatment) or to delayed (elective) CV with a randomization ratio of 2:3. The ratio is balanced to favour delayed CV with the a priori assumption that up to one in three patients in the delayed CV group may require expedited CV^6,20^ (i.e. before the intended waiting period of 5–9 days) for symptom relief. Randomization is performed in site-specific blocks with the intention of balancing patient randomization if the number of patients is low in any given participating centre (block size is blinded until the end of the trial patient enrollment). Randomization is performed through a dedicated website accessible by centre-specific user IDs and passwords. The group allocation is not blinded.

### Rhythm control and anticoagulation strategy in the acute rhythm control group

Patients randomized to acute rhythm control undergo CV in the ED. Electrical and medical CV are both viable options in the study, and the decision is made by the treating physician. If the first method fails (for example pharmacological CV), another can be adopted. Anticoagulation is initiated in accordance with the ESC guidelines.^9^ Patients are followed up and discharged from the ED following the standard procedure. All participating patients are scheduled an appointment at a cardiologic outpatient clinic within 5–9 days after the ED visit. The appointment time is scheduled before discharge or, at the latest, on the following working day.

### Rhythm control and anticoagulation strategy in the delayed rhythm control group

Patients randomized to delayed rhythm control are discharged immediately after their anticoagulation status has been secured and adequate rate control (heart rate <110 bpm) is ensured. The effectiveness of pre-existing anticoagulation therapy is verified (adherence to the use of direct oral anticoagulants [DOACs] or by verifying INR values for warfarin users) and patients with no prior anticoagulation are initiated on a DOAC regardless of the CHA_2_DS_2_-VASc score. However, if a patient experiences spontaneous return to SR before discharge and has very low thromboembolic risk (CHA2DS2-VASc = 0), no anticoagulation is initiated. Before discharge from the ED, or at latest the following working day, patients are scheduled for an appointment at a cardiologic outpatient clinic within 5–9 days, which includes the delayed (elective) CV. If the patient feels that the rhythm has spontaneously restored to SR before the planned CV, an ECG is taken to confirm this event on the next working day, at the latest.

The patient is referred to acute CV in the ED as soon as possible (with a transoesophageal echocardiography [TEE] guided CV for patients without previous anticoagulation therapy for at least 3 weeks, which has been shown to be safe^21,22^) if the patient randomized to delayed rhythm strategy feels that a more expedient CV is called for due to unbearable symptoms. All patients without previous anticoagulation for at least 3 weeks are advised of this option before consenting to the trial, including the possibility of TEE-guided CV. Patients also have the option to consult research personnel during office hours or go to the ED if the symptoms are unbearable during night-time or during weekend.

Patients are advised to seek acute medical care directly if they suspect that AF or AFL has recurred, or if they otherwise require urgent evaluation and/or care, regardless of whether study personnel have been contacted. This recommendation applies from discharge from the ED up until the first-month ECG control. If arrhythmia symptoms persist for a few hours and AF or AFL is recorded, the patient is referred to ED for implementation of acute rhythm control. If rate control is selected as the preferred strategy by the treating cardiologist at any point in the study, the ECG is, nevertheless, recorded at the earliest possible opportunity.

### Patient management in both groups

All patients visit cardiological outpatient clinic within 5–9 days after discharge from ED where their clinical condition and treatment is revised according to contemporary ESC guidelines.^9^ All patients are interviewed by a research nurse and a cardiologist to verify their prevalent health status. Briefly, all major contributing conditions for AF/AFL are screened for, and treatment strategy (rhythm or rate control and anticoagulation therapy) is revised if needed. Anamnestic information of possible shorter arrhythmia episodes not leading to healthcare visits and subsequent documentation are also recorded. Symptom severity is characterized using the European Heart Rhythm Association (EHRA) symptom scale^23^ and a standardized questionnaire covering several different aspects of AF-related symptoms.^24^ Relevant comorbidities, like obstructive sleep apnoea, are also screened using required additional diagnostics. Comprehensive transthoracic echocardiogram is performed for all subjects at the outpatient visit. Data on laboratory values, including additional relevant measurements such as thyroid hormone levels if not obtained during the ED visit, are collected. Information on CV procedures—including the number of prior CVs, methods used, success rates, and complications—is also recorded. If more aggressive rhythm control therapies are needed (antiarrhythmic medication by flecainide, amiodarone or dronedarone, or catheter ablation), initiation of the therapy is withheld for the first month to ensure unbiased estimate of rhythm status during the follow-up time. However, if aggressive rhythm control is indicated due to e.g. unbearable symptoms caused by repeatedly reoccurring atrial fibrillation or flutter paroxysm with the need for more than three CVs before the end of the first month after the outpatient clinic visit, it is allowed for in the protocol. If rate control is preferred as treatment strategy (patient asymptomatic), this will be implemented thereafter.

In the elective group, CV is performed during the visit to restore sinus rhythm if AF or AFL has not converted spontaneously by that time. Adequate protection against cardioembolic events is ensured by verifying that therapeutic anticoagulation has been maintained for at least 3 weeks prior to CV, or by TEE if adequate prior anticoagulation is not ensured^9^ (an option the patients are informed before enrollment). TEE prior to delayed CV in patients without adequate prior anticoagulation was incorporated into the protocol to ensure patient safety and to allow accurate assessment of the primary and secondary endpoints. If a thrombus is found in TEE, CV is delayed for at least 3 weeks to ensure adequate anticoagulation, and a new TEE is performed before CV to ensure resolution of the thrombus. The decision of long-term anticoagulation is made according to the ESC guidelines.^9^ Method of CV is selected by the treating cardiologist. If the first method fails (for example pharmacological CV), another can be adopted.

### Data collection and follow-up

Each patient completes a form requesting full disclosure of medical status and history in the ED, and this information is verified using electronic health records and written medical records which cover all prior specialized healthcare visits and the patients’ current medications. Furthermore, ED data on patient status (including haemodynamic parameters and laboratory values), received medical treatments and additional diagnostics are also collected.

After the outpatient clinic visit, a standard 12-lead ECG is recorded for all patients after 4 weeks (closest working day) from cardiology outpatient clinic visit. The date for this ECG is predefined so that the rhythm status is measured as it is at that date. In addition, patients are asked to fill in a symptom severity questionnaire also disclosing possible shorter arrhythmia episodes (not captured by ECG recordings) occurring during this time. A standard 12-lead ECG is also repeatedly recorded 3 months after the outpatient clinic.

All patient health records, and the Finnish Care Register for Healthcare are reviewed 1 and 3 months after the outpatient visit, as well as at 1 and 5 years after enrollment. Data on especially subsequent visits to ED due to AF/AFL and all other visits to specialized healthcare including major adverse cardiac and cerebrovascular events are recorded.

### Primary and secondary end-points

The primary end-point in this study is the presence of sinus rhythm verified by ECG on a predefined date 4 weeks after the visit to the outpatient clinic. The primary analysis will follow the intention-to-treat principle, comparing randomized groups irrespective of spontaneous conversion or expedited CV in the delayed group. A per-protocol analysis will be conducted as a sensitivity analysis in accordance with recommendations for non-inferiority trials.^25^ Early unplanned CV (0–4 days after ED visit) due to unbearable symptoms in the delayed rhythm control strategy is considered a prespecified intercurrent event and leads to exclusion from the per-protocol analysis. This exclusion is performed to study the non-inferiority of successfully delaying the CV by at least 5 days from presentation to ED. Patients with missing end-point data are excluded from the analyses, and the number of patients with missing data is reported. Secondary end-points include the overall recurrence of AF or AFL, the number of required CVs and duration of ED stay, the need to use antiarrhythmic drugs within the first month, unplanned CV rate within the first week due to unbearable symptoms (elective approach), cardiovascular events—including events monitored for safety, and quality of life. The details of the end-points are listed in *Table 2*.

**Table 2:** End-points of the randomized controlled AFFELECT trial

Outcome measure	
PRIMARY OUTCOME	
Sinus rhythm	Prevalence of sinus rhythm in the treatment arms measured by electrocardiography at 1 month. The ECG is taken on a prespecified day aiming for the closest working day 4 weeks after the outpatient clinic visit (4–5 weeks after original ED visit).
SECONDARY OUTCOMES	
Number of cardioversions	First week after randomization (before preplanned outpatient clinic visit) One month after out-patient clinic (before control ECG) Overall (from randomization to 1 month control ECG)
Unplanned CVs in delayed cardioversion group	The number of subjects needing unplanned cardioversion before preplanned outpatient clinic visit (within four days after randomization) due to medical reasons or due to subjectively perceived unbearable symptoms (EHRA III or IV).
New initiation of antiarrhythmic drugs (AAD)	Unplanned initiation of AADs at any point during the first month First week after randomization (before preplanned out-patient clinic) One month after outpatient clinic (before control ECG) Overall (from randomization to 1 month control ECG)
Proportion of patients with significant change in treatment strategy	The proportion of patients in whom the treatment strategy is changed from rhythm control to rate control due to low symptom burden, as evaluated at first cardiology outpatient clinic visit in accordance with ESC guidelines.
Hospitalization due to cardiovascular causes^a^	First week after randomization (before preplanned out-patient clinic) One month after outpatient clinic (before control ECG)
Quality of life	Quality of life as assessed by standardized questionnaires (AFEQT, EHRA-scale) First week after randomization (before preplanned out-patient clinic) One month after outpatient clinic (before control ECG)

^a^Serious adverse events are screened for during the first month of the study (before control ECG). Serious adverse events include thromboembolic events (ischaemic strokes and other thromboembolic events), hospitalization for decompensated heart failure or for serious arrhythmic events (sudden cardiac death or events leading to cardiopulmonary resuscitation) and hospitalization due to any cardiovascular complication or disease.

In addition, end-points are evaluated at 3 months and 5 years after the clinic visit, including recurrence of atrial arrhythmias at both time points and incidence of permanent AF/AFL at 5 years. Symptom severity is assessed up to one month, but not at 5 years.

### Interim analysis

The AFFELECT trial is deemed a low-risk trial and no independent safety monitoring board was established based on prior evaluation and ethical committee decision. However, continuous monitoring for serious adverse events (thromboembolic events and hospitalizations due to heart failure) is applied.

A statistical interim analysis for serious safety events was performed in March of 2023 by an independent statistician from Tampere University, 5 weeks after the randomization and obtaining first month follow-up data of 200 patients comparing the risk of cardiovascular hospitalizations blinded to the group allocation. No statistically significant association was found, and the study was continued as planned.

### Sample size calculations and considerations

The power calculation indicated that 500 randomized patients are required to ensure 90% power (one-sided alpha level of 0.025) to reliably demonstrate non-inferiority, should the elective treatment prove inferior with respect to the primary end-point. The experimental treatment (elective approach) will be considered inferior if the lower bound of the 95% confidence interval for the difference in sinus rhythm prevalence (elective minus acute) falls below −10%. The calculation is based on the assumption that the prevalence of sinus rhythm in the control population is 90%. A 10% margin in sinus rhythm prevalence between the treatment groups is deemed clinically acceptable, as the prevalence of sinus rhythm can fluctuate daily among patients, and a variation of less than 10% is unlikely to affect the long-term prognosis of the participants. If patients were randomized into two equal groups, the total sample size would be 380 patients. However, a 2:3 randomization ratio was chosen to conservatively account for the anticipated occurrence of unplanned early CV in the delayed strategy group, which would lead to exclusion from the per-protocol population, as patients were informed about this possibility prior to randomization. For every patient randomized to the acute CV treatment group (at least 190 patients), three patients will be randomized to the elective CV treatment group (at least 285 patients). This randomization ratio accounts for the possibility that up to one-third of patients may request more urgent CV after randomization (with no unplanned CVs for unbearable symptoms the minimum number of patients would be thus 190 + 190 patients). This assumption was based on previous findings from the FinFib study, which indicated that up to one-third of patients undergoing emergency CV experience symptoms severe enough to interfere with daily life (EHRA Class III–IV)^6,20^ and therefore they might need an earlier CV. Considering the potential loss of patients to follow-up, the study aims to recruit approximately 5% (25) more participants than the minimum required by the power calculations (475 patients), resulting in a total of 500 patients.

Statistical analyses will be performed by the investigators in collaboration with an independent statistician from Tampere University who is not involved in data collection. Established statistical methods are employed to compare the differences between groups. χ^2^ testing is used to compare the distributions of categorical variables, while parametric testing methods, as *t*-test, are applied to normally distributed continuous variables. Non-parametric testing methods are used for variables not following normal distribution.

## Discussion

Early CV in the ED has long been the standard approach to managing recent-onset AF/AFL. However, given the high recurrence rate of AF and the growing number of affected patients, this strategy places a significant burden on emergency services, where resources are often stretched and patient turnover is high.^1–3,5^ The AFFELECT trial introduces a novel approach to the management of acute AF/AFL by challenging the long-standing practice of immediate rhythm control in the ED.

The AFFELECT trial proposes that rhythm control can be safely deferred for 5–9 days in clinically stable patients, shifting care from the ED to elective cardiology units. This approach allows for spontaneous conversion in many cases^16^ and facilitates a more measured, patient-centred decision-making process regarding CV. The clinical comparison focuses on overall sinus rhythm prevalence at 4 weeks and the total number of CV procedures required to achieve this outcome in each strategy. This reflects the real-world decision faced in the ED and promotes a more individualized, resource-conscious, and clinically deliberate pathway that better aligns with practical clinical management.

Treating haemodynamically stable patients in dedicated elective cardiology units rather than crowded EDs could offer several advantages. It may substantially reduce the number of CVs performed, lower healthcare costs, and prevent repeated unnecessary ED visits. Moreover, concentrating the management of these patients in specialized cardiology settings enables more structured follow-up, coordinated decision-making, and comprehensive rhythm control strategies, which are associated with a reduction of morbidity and mortality in selected patients.^26^ If successful, this paradigm could meaningfully redefine the standard of care for acute AF/AFL, aligning patient safety, clinical efficiency, and sustainable healthcare delivery.

## Conclusion

The AFFELECT trial will demonstrate whether the delayed approach of rhythm control in recent-onset AF or AFL is non-inferior compared to standard treatment with respect to long-term maintenance of sinus rhythm. In this scenario, the number of ED visits and CV procedures could decrease significantly, alleviating the burden on emergency services, while directing the treatment of AF/AFL patients to specialized units focused on AF and AFL care.

## Data Availability

Data will be made available upon reasonable request to the chief investigator, subject to applicable data protection regulations and pending the approval of the study steering committee.

## Lead author biography

**Dr Jani Rankinen** is a cardiologist at the Heart Hospital of Tampere University Hospital (TAYS), Finland. His professional interests focus on interventional cardiology, in which he is currently specializing. He holds a PhD in Medicine from the University of Tampere and works as a postdoctoral researcher alongside his clinical duties, with a primary research focus on sudden cardiac death.
